# Durability Improvement of Biocemented Sand by Fiber-Reinforced MICP for Coastal Erosion Protection

**DOI:** 10.3390/ma15072389

**Published:** 2022-03-24

**Authors:** Md Al Imran, Kazunori Nakashima, Niki Evelpidou, Satoru Kawasaki

**Affiliations:** 1 Graduate School of Engineering, Hokkaido University, Sapporo 060-8628, Japan; 2 Faculty of Engineering, Hokkaido University, Sapporo 060-8628, Japan; k.naka@eng.hokudai.ac.jp (K.N.); kawasaki@geo-er.eng.hokudai.ac.jp (S.K.); 3 Faculty of Geology and Geoenvironment, National and Kapodistrian University of Athens, 15784 Athens, Greece; evelpidou@geol.uoa.gr

**Keywords:** MICP, jute fiber, durability, soil improvement, biocement, fiber reinforcement, coastal erosion protection

## Abstract

Soil improvement via MICP (microbially induced carbonate precipitation) technologies has recently received widespread attention in the geoenvironmental and geotechnical fields. The durability of MICP-treated samples remains a critical concern in this novel method. In this work, fiber (jute)-reinforced MICP-treated samples were investigated to evaluate their durability under exposure to distilled water (DW) and artificial seawater (ASW), so as to advance the understanding of long-term performance mimicking real field conditions, along with improvement of the MICP-treated samples for use in coastal erosion protection. Primarily, the results showed that the addition of fiber (jute) improved the durability of the MICP-treated samples by more than 50%. Results also showed that the wet–dry (WD) cyclic process resulted in adverse effects on the mechanical and physical characteristics of fiber-reinforced MICP-treated samples in both DW and ASW. The breakdown of calcium carbonates and bonding effects in between the sand particles was discovered to be involved in the deterioration of MICP samples caused by WD cycles, and this occurs in two stages. The findings of this study would be extremely beneficial to extend the insight and understanding of improvement and durability responses for significant and effective MICP treatments and/or re-treatments.

## 1. Introduction

Coastal erosion is currently a pressing issue throughout the world. Due to the limitations of existing countermeasures, it is critical to create alternative sustainable and environmentally acceptable solutions for preventing coastal erosion. Biostabilization techniques, such as MICP (microbially induced carbonate precipitation), have received a lot of interest from researchers all over the world as alternative countermeasures. In addition, significant attention has been paid to bioinspired soil improvement as an innovative and pragmatic way to advance soil and ground conditions. MICP as a coastal erosion control approach is still a relatively new concept. While numerous studies [1,2,3] have indicated that MICP-treated soil improves the strength of the samples, there is no information on the known association between fiber-reinforced soil strength and erodibility when utilizing the fiber-reinforced MICP approach. As a result, erosion protection—especially for coastal areas—must be considered separately from the perspectives of improvement of strength and durability characteristics. Microbially induced carbonate precipitation (MICP) has been identified as a viable and effective strategy for soil improvement among the various bio-mediated soil improvement technologies in recent years. During the MICP immobilization and strength improvement, the microbial urease hydrolyzes the urea [CO(NH_2_)_2_] and generates ammonium and carbonate ions, raising the pH [4,5]. MICP treatment clearly alters the soil from a loose to an aggregated form (i.e., soft rock-like material), showing that the calcium carbonate bonding of soil particles reduces particle flexibility, mobility, and detachability, which has a direct impact on the durability of the MICP-treated samples. Many recent experiments, in contrast, have established the mechanism of calcium carbonate deposition and soil strength improvement after curing the MICP-treated samples under various environmental conditions [6,7]. The goal of this study was to determine the viability of the fiber-reinforced MICP approach as a unique alternative and effective countermeasure for coastal erosion protection, by determining how it works or can be applied practically using the proposed coastal erosion protection method [8]. Furthermore, the goal of this research was to determine appropriate or possible conditions and methods for creating artificial beach rocks for coastal erosion prevention in Greece and other Mediterranean countries via a cost-effective, environmentally friendly, and long-term approach. In this study, natural jute fiber was also used as a sophisticated bio-mediated technology to test the samples’ strength and durability. The findings of this study could constitute a precious source of information for viable commercial applications for coastal erosion protection and other bioengineering applications in the future. MICP-treated soil has previously been observed to have non-uniform calcium carbonate precipitation and brittleness failure behavior [9]. Earlier studies also revealed that the MICP-treated soil tends to rupture on a low axial strain during unconfined and triaxial compression tests, and the axial stress fails quickly after the peak stress, since most of the calcium carbonate precipitates indiscriminately [10]. In one study, this carbonate precipitation process took place next to the specimen column’s effluent, and hampered the biocementation process for deeper scale, leading to the development of less durable material [11].

To address this problem, experiments have been undertaken to increase the ductility and toughness of sand after curing MICP sand [12]. Coastal erosion protection is gaining popularity among geotechnical engineers as one of the many potential applications of fiber-reinforced MICP-treated biocemented sand. Rainfall, evapotranspiration, and other climatic variations are all common in exposed coastal areas over several decades. Another major weathering agent is wet–dry (WD) cycles, which have been a major concern in geotechnical and geological engineering sectors—particularly for coastal erosion control [13]. The surface experiences periodic WD cycles as a result of direct interaction with the environment during (1) diurnal shifts in rainy and/or sunny weather, and (2) subsequent rainfall evapotranspiration processes [14]. Several studies have demonstrated that the WD cycles generate significant, irreversible deformations in the fabric of geomaterials [15]. For example, it has been discovered that the existence of swelling minerals causes temporal alteration to the soft or targeted soils during WD cycles, resulting in desiccation cracking, volumetric changes, and shrinkage [16].

The WD procedure changes the microstructure of soil aggregates, resulting in reductions in shear strength and compressibility. The most observed degradation mechanisms in sedimentary materials include fracture energy reduction and chemical corrosive activity. The mechanical characteristics of calcareous rocks and sandstone materials have been shown to be considerably deteriorated by the WD process in several studies [17]. Because the reactions of MICP-treated soils (cemented by CaCO_3_) are generally comparable to natural carbonate and/or sandstone sediments [18], the treated samples are more likely to deteriorate in a similar way when exposed to the cyclic WD process repeatedly. It is worth noting that there is a scarcity of data on the weathering effects of MICP treatment under repeated WD cycles. Therefore, prior to field applications, a performance assessment in terms of durability is required and recommended.

## 2. Materials and Methods

### 2.1. Fiber Material Used in This Study

Previous studies have shown that jute fiber, with a specific length and composition, can increase the strength and durability of MICP-treated samples significantly [19,20]. Furthermore, jute fibers have a high initial modulus, consistent tenacity and tensile strength, high stiffness, and a low percentage of elongation upon breakage, all of which have led to their widespread usage in soil restoration, and motivate their use in this work. This study employed locally available (100% natural) jute fiber that had not been chemically treated. “DCM Homac Co., Ltd.” in Sapporo, Japan provided the jute fiber, which was collected by “Hayase Industries, Ltd.” in Japan. Scanning electron microscopy images were used to analyze the microstructure of the jute fiber.

Table 1 shows the essential characteristics of the jute fiber employed in this investigation. The efficiency of fibers employed for soil improvement is strongly dependent on attributes such as fiber type, fiber length, and fiber ratio, as revealed in previous studies. Jute fibers were cut at three different lengths (5 mm, 15 mm, and 25 mm) and mixed with Mikawa sand particles at various percentages (content) by weight (0.5%, 1.5%, 3%, 5%, 10%, and 20%). Previous experimental results [9,21] showed that 15 mm long fibers length contributed towards samples’ strength improvement; thus, 15 mm fiber length was chosen for durability analysis in this study. Before mixing with the sand and MICP treatment, all of the jute fibers utilized in this study were dried for 24 h at 60 °C.

### 2.2. Used Bacteria and Soil Properties

*Micrococcus yunnanensis* (hereafter referred to as G1) was isolated from the coastal regions of Porto Rafti, Greece, and was employed in this investigation [22]. The key characteristics of this bacterium are elevated urease activity with salt-tolerant prominence, the ability to survive for long periods of time at different temperatures and pH levels, and the capacity to thrive in nutrient-deficient situations [23]. For the selected bacterial species, liquid ZoBell2216 medium was employed as a culture solution. Hi-polypeptone (5.0 g/L), FePO_4_ (0.1 g/L), and yeast extract (1.0 g/L) were used to dissolve the culture medium.

**Figure 1:**
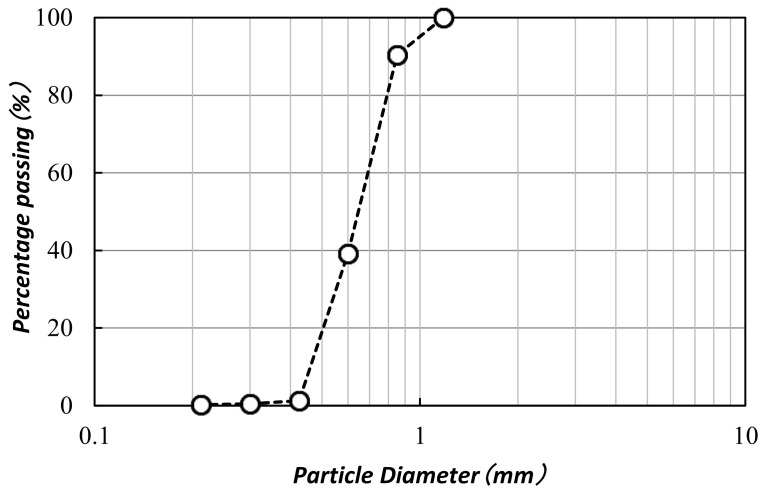
The granulometry of the Mikawa sand used in this study.

The ingredients were combined with the artificial seawater and kept at a pH of 7.6–7.8. The bacterial cells were pre-cultured in a shaker at 160 rpm for 24 h at 30 °C, using the ZoBell2216 medium. After that, the pre-cultured bacterial cells were shifted (1 mL) to 100 mL of viable ZoBell2216 medium and incubated at 30 °C and 160 rpm. For the MICP procedure, a drawn-up bacterial culture solution was used. During the cultivation, the bacterial cell growth (OD_600_) was monitored using a UV–Vis spectrophotometer (V-730, JASCO Corporation, Tokyo, Japan), and the urease activity (1.5 ± 0.3 U/mL) was measured by means of the indophenol method [23]. Commercially available “Mikawa” sand (commercially available at standard lab grade with uniform sizes, Tokyo, Japan) was used in this study. The highest and lowest dry densities of the “Mikawa” sand were 1.476 and 1.256 g/cm^3^, respectively; the particle density was 2.66 g/cm^3^, and mean diameter was 870 μm. The granulometry of the “Mikawa” sand is shown in Figure 1. The sand was dried up in an oven drier at 110 °C for 24 h before being used in the MICP process.

### 2.3. Sample Preparation for Cyclic Wet–Dry Tests

**Figure 2:**
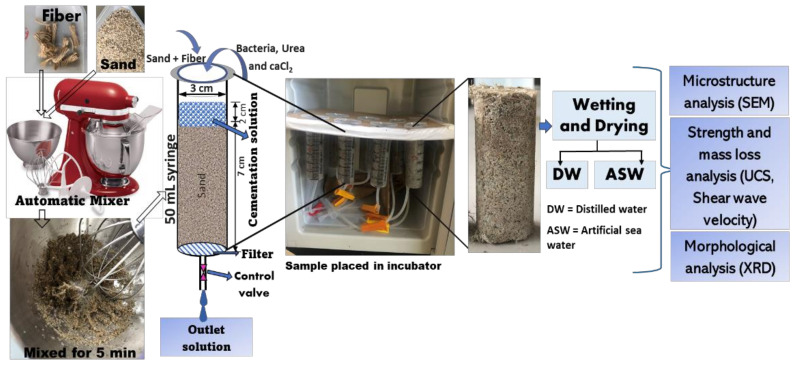
Experimental procedures.

Figure 2 depicts the MICP’s proposed materials and test setup. Dry “Mikawa” sand (75 ± 5 g) was placed in a 50 mL standard syringe tube (diameter 3 cm, height 10 cm). By giving a hammer shock to each layer of the sand, oven-dried samples (as stated before) were compressed into three layers. The bottom section of each column was covered with a lab-grade filter paper. By using an automated mixer (kitchen aid 9KSM160 series, Tokyo, Japan) with varied lengths and fiber contents, each sand column was packed with consistently distributed jute fiber. During the mixing procedure, 10 mL of deionized water was introduced in order to neutralize the existing electrostatic charge of the fiber and sand grains, ensuring that the fiber was distributed uniformly throughout the soil matrix.

Next, 12 mL of bacterial culture liquid (ZoBell2216E) was injected into the soil matrix from the top of the syringe, and the excess solution was flushed-out at a regulated pace for bacterial stabilization (~2 h). Then, 16 mL of cementation solution (30.0 g/L urea, 55.0 g/L CaCl_2_, and 3.0 g/L nutritional Bacto broth) was injected into the samples during the later injection phase. The injected solution was maintained ~2 mL above the sand’s surface in order to maintain a completely saturated state. For 14 days, the produced samples were stored at 30 °C in an incubator. Prior to completing the wet–dry (WD) cycle testing, all measurements were taken after the samples were removed from the syringes and dried.

**Figure 3:**
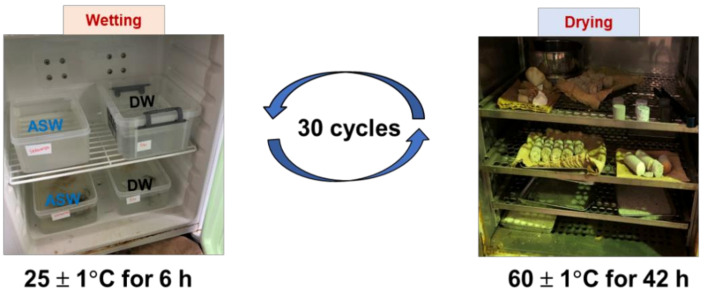
Cyclic wet–dry testing procedure used in this study.

The wet–dry (WD) cycle tests were carried out as suggested by the ASTM D559-03 (2003) standard method, using distilled water (DW) and artificial seawater (ASW) for standard comparison, and mimicking the real field conditions, in the pursuit of coastal erosion protection. The testing conditions for durability analysis are presented in Table 2 (DW cases) and Table 3 (ASW cases). Specimens were completely immersed in the distilled water in room temperature (25 ± 1 °C) for 6 h throughout the wetting procedure. Prior to the next WD cycle, the saturated specimens were put in the oven at 60 ± 1 °C for a minimum of 42 h, and when the weight of the specimens remained consistent, their dry weight was noted (Figure 3). The effects of WD cycles were investigated using the following measurements after specimens were subjected to a maximum of 30 continuous WD cycles: the total number of cycles was used in calculating the weight loss and S-wave velocity of the specimens, scanning electron microscopy (SEM; Miniscope TM3000, Hitachi, Tokyo, Japan) was used to study the morphology of the precipitated CaCO_3_ crystals before and after the WD tests, and X-ray diffraction (XRD; MiniFlex^TM^, Rigaku Co., Ltd., Tokyo, Japan) analysis was used to assess the polymorphism of the precipitated CaCO_3_.

## 3. Results and Discussion

### 3.1. Physical Changes and Weight Loss

**Figure 4:**
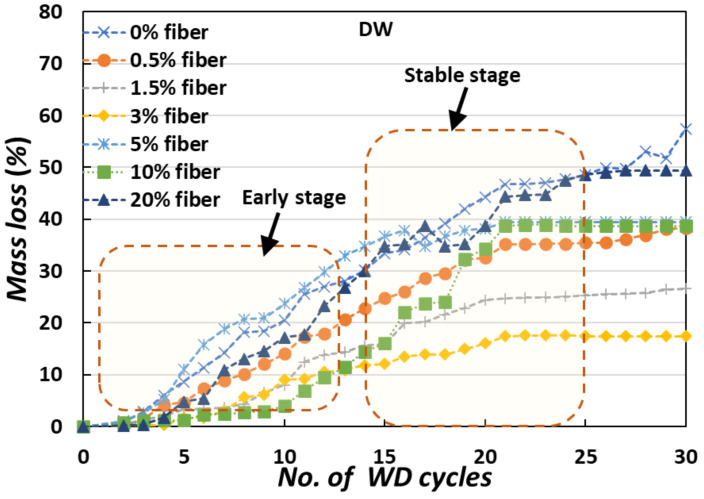
The average weight loss of the MICP-treated samples exposed to cyclic WD treatments (DW cases).

The weight loss from the specimens was used to assess the physical degradation of the MICP-treated samples during the cyclic testing. After each cycle, the weight loss was precisely monitored using distilled water (DW) and artificial seawater (ASW) at a consistent temperature. The weight loss of the specimens exposed to 30 cyclic WD treatments is shown in Figure 4. In the case of 0% fiber-treated MICP specimens, weight loss was found to be greater than 50% after 30 WD cycles. The weight loss tendency decreased as the amount of added fiber was increased, and in 3% fiber-treated MICP specimens, the weight loss was just 20% (DW cases) and 30% (ASW cases).

**Figure 5:**
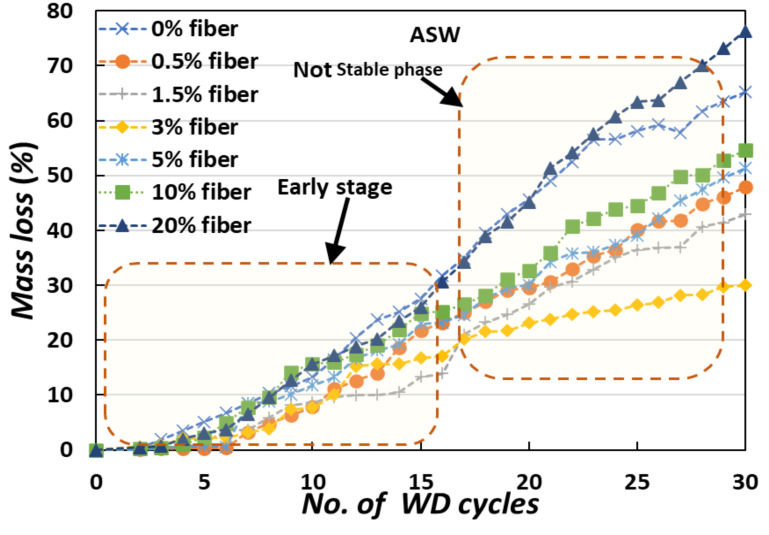
The average weight loss of the MICP-treated samples exposed to cyclic WD treatments (ASW cases).

In the case of DW, severe weight loss occurred at an early stage. After 20 cycles, the specimen became more stable, and severe weight loss did not occur. Without the addition of fiber, the weight loss occurred rapidly (around 40% after 20 cycles), but with the addition of fiber, weight loss occurred gradually (around 20% after 20 cycles). The rate of weight loss steadily decreased for several more WD cycles (Figure 4), and tended to reach equilibrium. In the case of ASW (Figure 5), severe weight loss occurred continuously. Without the addition of fiber, the weight loss was around 60% after 20 cycles, but with the addition of fiber, weight loss occurred gradually (around 25–30% after 20 cycles) in the treated specimens [24,25].

### 3.2. Strength Deterioration Ratios

**Figure 6:**
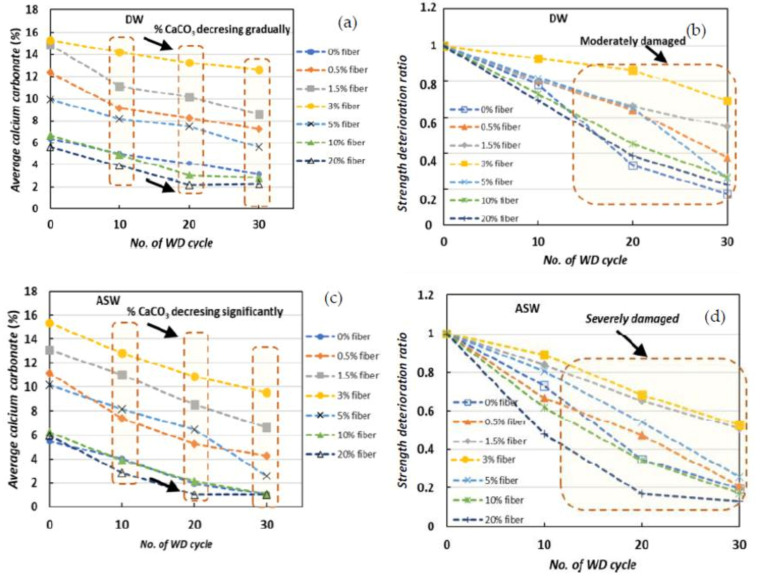
The average calcium carbonate (%) of the samples treated with (**a**) DW and (**c**) ASW, and the strength deterioration ratio in (**b**) DW and (**d**) ASW after the exposure to cyclic WD tests.

From the results, the WD cycles were clearly shown to have a substantial impact on the physical and mechanical characteristics of the fiber-treated MICP samples. Strength deterioration ratios (SDRs) were determined using the following equation to investigate the influence of fiber-reinforcement on the mechanical degradation (Equation (1)) of the samples:

(1) $$\mathrm{SDR}=1-\frac{{\mathrm{UCS}}_{\left(f\right)}}{{\mathrm{UCS}}_{\left(i\right)}}$$

where the compressive strength by the end of the cyclic treatment is represented by UCS_(*f*)_, while the compressive strength of the primary MICP-treated samples is represented by the UCS_(*i*)_. According to the results (Figure 6a), the average CaCO_3_ was steadily decreasing, and substantial deterioration was observed in all cases during the first stage (up to 10 WD cycles). In the case of DW, the SDR value was not significant in the early stages (up to 10 WD cycles), but with more cyclic treatments (up to 30 WD cycles) the SDR value decreased significantly (Figure 6b). In the case of ASW, the SDR values decreased significantly when the calcium carbonate concentration decreased (Figure 6c). This pattern suggests that the significant carbonate deposition in ASW preserves the MICP responses during the WD cycles. Fiber-treated MICP specimens achieved a high precipitation content in a stable form, which could strengthen the principal carbonate bonding connections and provide strong resistive forces during the development of fatigue stress. Furthermore, these robust connections are able to withstand the suspension and corrosion caused by the WD process, resulting in an increase in SDR values (Figure 6d).

However, from the cyclic WD tests (using the DW and ASW), it was clearly indicated that only MICP-treated samples underwent acute mechanical deterioration (around 60%) during the ASW tests, and that the addition of fiber significantly improved the strength deterioration ratio (by around 40–50%). This overall investigation (Figure 6) suggested that the mechanical deterioration occurred more prominently early in the WD process (ASW) compared with that in the later phases (DW), and the addition of fiber could significantly improve the durability of the MICP-treated samples.

### 3.3. Measurements of Shear-Wave Velocity

**Figure 7:**
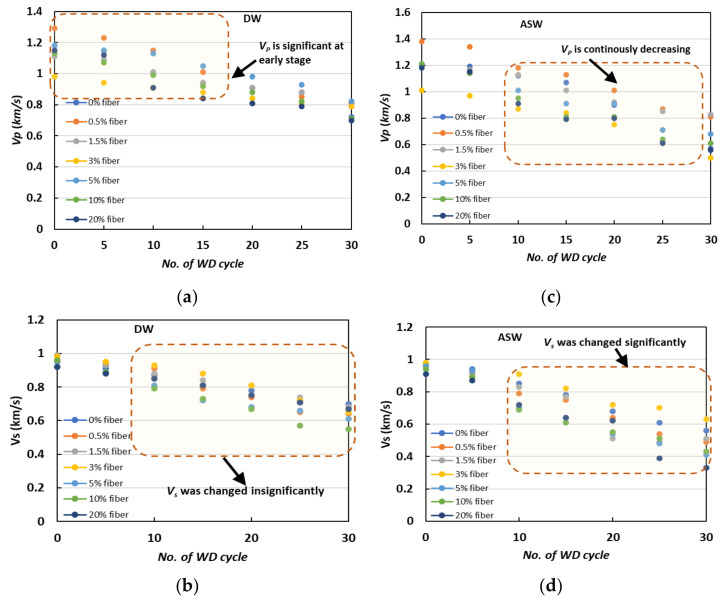
Variations in the shear-wave velocity of specimens under exposure to cyclic WD tests: (**a**) P-wave velocities in DW; (**b**) S-wave velocities in DW; (**c**) P-wave velocities in ASW; (**d**) S-wave velocities in ASW.

The samples were monitored using S-wave and P-wave velocity to better understand the changes in their mechanical behavior, and the findings are shown in Figure 7. Since the P-waves and S-waves can only be transmitted through solids, the deposited calcium carbonate at the grain contact point had the greatest impact on the P-wave velocity (Figure 7a) and S-wave velocity (Figure 7b) (not through soil pores) [26,27].

In both DW (Figure 7a,b) and ASW (Figure 7c,d), the variance in velocity data was followed by a relatively similar tendency towards an increase in the number of WD cycles, regardless of the cementation levels. During the first few cycles (up to 10 WD cycles), a rapid drop was seen, followed by a smooth and progressive decrease. These rapid decreases in P-wave and S-wave velocity are consistent with those reported in weight loss, and could be related to the corrosion of grain connections caused by the WD process’s deterioration. By contrast, the average velocity declined between 5 and 15 WD cycles, with values of 0.08–0.12 km/s, implying modest damage at the later stage, while severe damage occurred during the ASW cycles. This observation is remarkably similar to the observed %CaCO_3_ and strength deterioration ratio described in earlier sections.

### 3.4. Microstructural Analysis (SEM and XRD) of the Treated Samples

**Figure 8:**
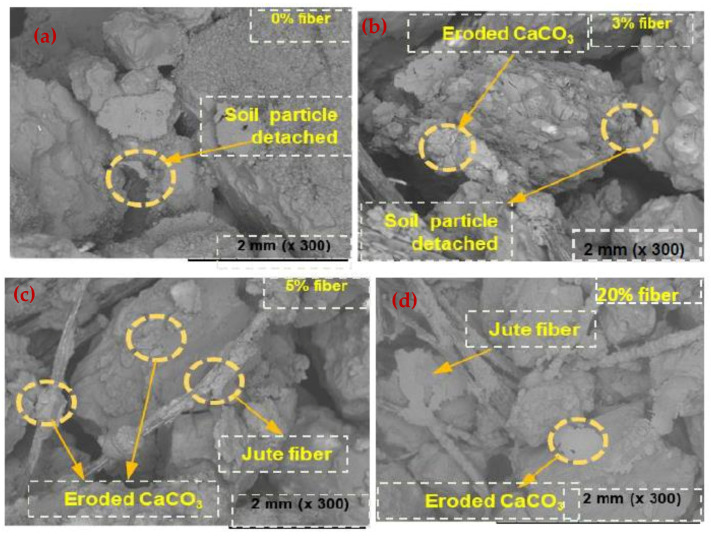
SEM images (DW) of the specimens after exposure to WD cycles. (**a**) 0% fiber; (**b**) 3% fiber; (**c**) 5% fiber and (**d**) 20% fiber.

**Figure 9:**
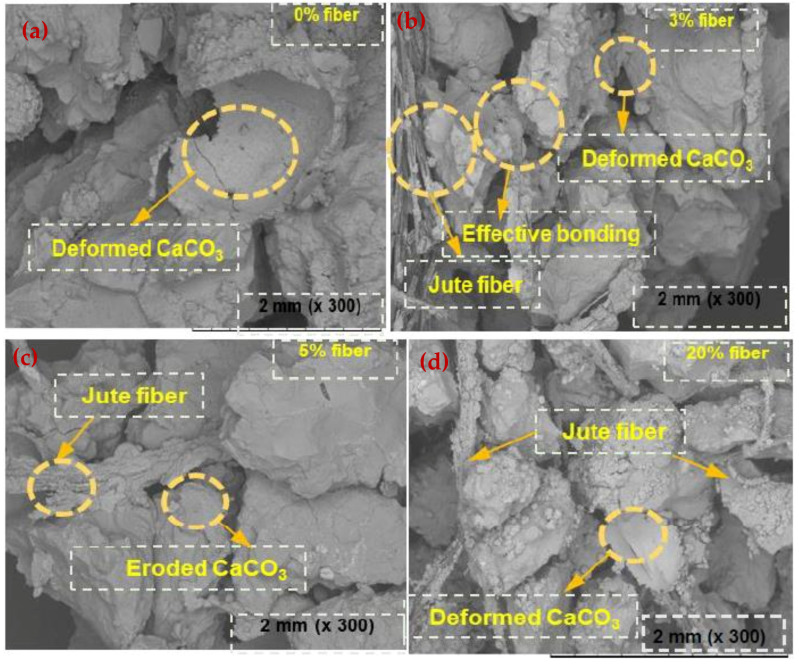
SEM images (ASW) of the specimens after exposure to WD cycles. (**a**) 0% fiber; (**b**) 3% fiber; (**c**) 5% fiber and (**d**) 20% fiber.

The SEM images showed that the soil structure was clearly impacted during the first few WD phases, but then tended to remain fairly constant (referred to as the stable phase) (Figure 8). When compared to the core of the specimens, the surface of the specimens showed more continuous deterioration (referred to as the unstable phase). The carbonate deposits on the surface were decreased and eroded. The generated microcracks were also observed to have eroded bonds, and the severity of the microcracks increased dramatically when the samples were treated with ASW (Figure 9).

When the MICP-treated specimens were dried, their inner relative humidity did not drop instantly but, rather, gradually [28], and their surface evaporated quickly, causing substantial negative stresses. Furthermore, because the samples’ core zone was tightly restricted, the produced fatigue stresses caused greater damage to the columns of the outer surface, resulting in the advancement of the macropores. Some biocemented specimens have been reported to have similar early surface degradation due to WD forces [29]. Additionally, the disparity among the heat expansion coefficients between the calcium carbonate and the soil material might be an added element that caused internal fatigue stresses to develop during temperature changes, resulting in carbonate bond degradation [30]. This is because the internal fatigue loads and deformation occurred in holes and microcracks during the carbonate cementation process. The long-term weathering of this kind of carbonate rock is caused by the disintegration of diagenetic calcite interactions at a time when calcites are filled with water for an extended period of time [31].

The effect of dissolution was believed to be insignificant in this investigation under ordinary conditions. When carbonate rocks were subjected to saturation, immediate debonding of depositional linkages (menisci-shaped calcite ensembles) occurred. The researchers also discovered that a single wetting procedure resulted in a significant reduction in compressive strength, which was especially noticeable in high-porosity calcareous cements [32].

### 3.5. Durability Characteristics and Mechanisms

**Figure 10:**
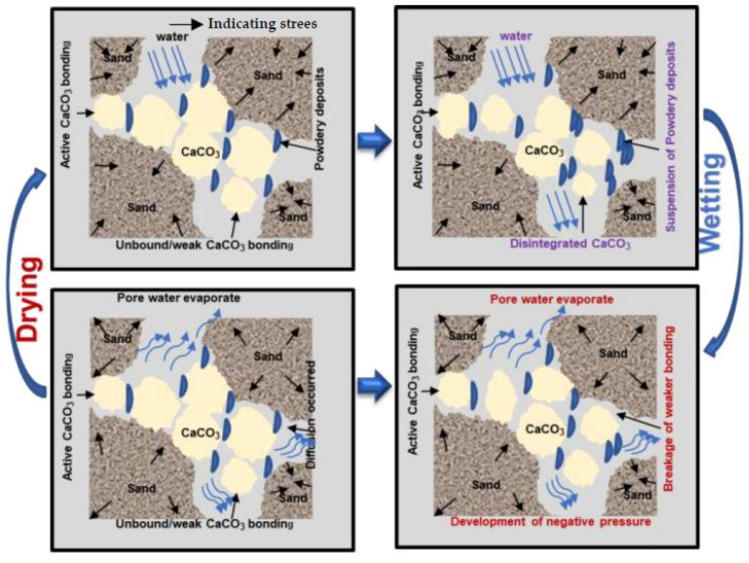
Schematic illustration of the deformation process of MICP-treated specimens after exposure to WD cycles (without the addition of fiber).

Calcium carbonate precipitated in a variety of forms during the MICP treatments, including primary bonds (active development at grain contact points), certain crystals and their accumulation (at the grain surface), and amorphous and powder-like deposits [21,28,31]. In fact, powdery linkages are frequently generated when carbonates are accumulated early in the crystallization process. Water enters the samples during the wetting process, causing the powdery granules to accumulate into a suspension (Figure 10). The mechanism of corrosion is similar to that of calcarenite rocks reported in earlier sections [8,10,32]. The soils treated with MICP have a porous texture. Relatively speaking, the subsequent stress imposed on the soil particles and disseminated to the links within the sample matrix specifically affects the calcium carbonate connections. The linkage would crack if the acting tensile stresses exceeded the extreme tensile strength of the existing carbonate bonds.

**Figure 11:**
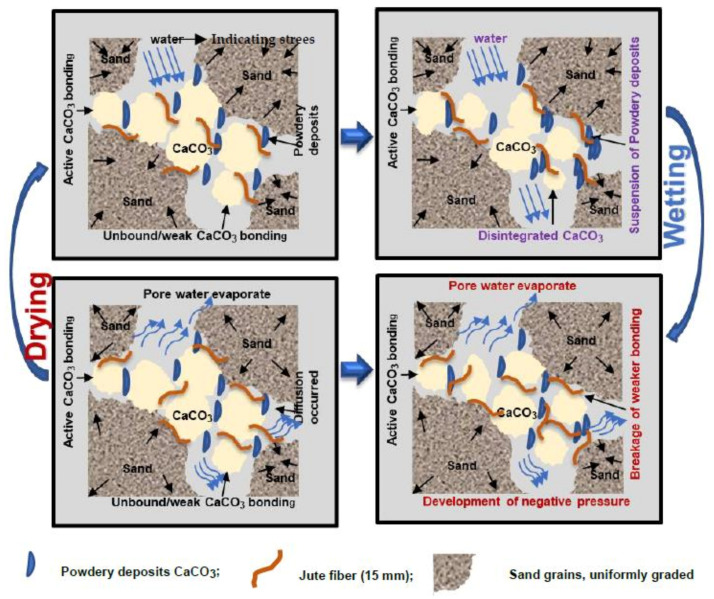
Schematic illustration of the deformation process of fiber-reinforced MICP-treated specimens after exposure to WD cycles. Position on the left.

When the soil samples were treated via MICP, a portion of the CaCO_3_ precipitation was only connected to the surface of particles that may not have linked to the sand particles and, thus, could simply be eroded during the WD cycles. During the early wetting, water diffused across the porous membrane and inundated the material, causing the powdery deposits fall into suspension and break down, leading to loss of particle contact and weight loss. The fiber functioned as a connector and source of tension between the soil particles and calcium carbonate, and improved the soil particle interaction. In MICP-treated samples with fiber, fibers throughout the samples absorbed the tension within the soil matrix because of the fiber–soil friction, efficiently improving the durability of the samples (Figure 11). However, erosion caused by the WD cycle could be alleviated by treating the soil with a substantial percentage of fiber-reinforcement and a high amount of calcium carbonate.

**Figure 12:**
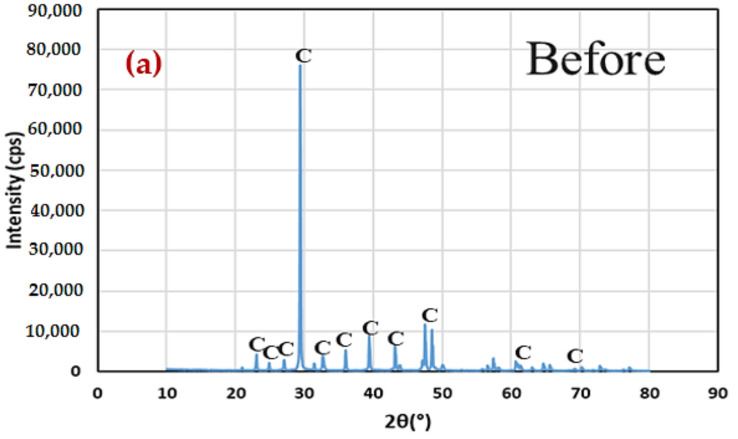

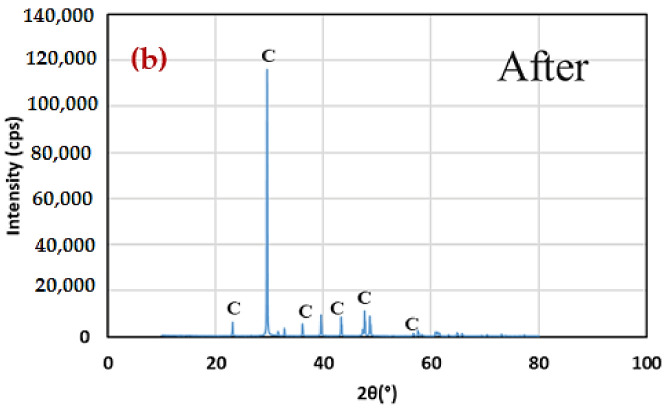
XRD results of the treated specimens exposed to WD cycles. (**a**) Before (**b**) After.

The findings of the XRD investigation are shown in Figure 12. The investigation was carried out to confirm the presence of phase transformations in carbonate deposits, as well as the deposition of other minerals and the dissolution of remaining minerals. The XRD findings showed that there were no visible alterations in mineral morphology during the WD trials with either DW or ASW. The observed peaks were equal to the rise in the number of cycles, implying that neither additional minerals were produced nor were phase transitions generated.

## 4. Conclusions

The purpose of this study was to investigate how the added jute fiber influenced the durability and strength of the samples biocemented using the MICP method, intended for application in coastal erosion protection using the proposed methodology [7]. Jute fiber was employed at six distinct levels in the study—including 0, 0.5, 1.5, 3, 5, 10, and 20% (by sand weight)—and three distinct lengths (5, 15, and 25 mm) for the treatment of the sand via the MICP technique. The following conclusions could be drawn from the findings of this investigation:

I. In both DW and ASW, cyclic wet–dry (WD) effects appear to have a negative impact on the mechanical and physical properties of fiber-reinforced MICP-treated samples. The dissolution of calcium carbonates and bonding effects between sand particles was discovered to be involved in the deterioration of MICP samples caused by WD cycles, and this occurred in two stages: short-term and long-term;
II. As a result of the suspension and degradation of the powdered carbonate accumulations, short-term disintegration occurred rapidly within the first couple of WD cycles in DW. After 15 WD cycles in ASW, long-term degradation was observed;
III The fatigue stresses generated during the WD cycle have the potential to break the primary bonds, affecting the surface of the specimens in particular. However, the addition of jute fiber improved the bonding effects and increased the aggregate stability and physical durability. Our findings also revealed that the MICP-treated samples are less vulnerable to WD cycle treatments (DW cases), i.e., more stable behavior in DW cases compared with that in ASW cases. However, the addition of fiber could significantly improve the strength and durability of the MICP-treated samples;
IV. Natural jute fiber was used in this work; nevertheless, the impacts of chemically treated jute fiber and fiber roughness (surface) were not thoroughly studied in terms of improvements in strength and durability. More research is needed for better understanding of the impacts of fiber-reinforcement on soil stabilization via the MICP process (considering chemical pretreatment of the fiber, fiber roughness, etc.)

## Figures and Tables

**Table 1:** The main characteristics of the jute fiber used in this research.

Fiber Name	Density	Range	Weight	Pattern	Moisture Content	Complexion
Jute	2 mm	510 m	900 g	Roll	3.4%	Golden-brown

**Table 2:** Testing conditions used in this study for durability analysis (DW cases).

Testing Cases	Fiber Content ((%) mm)	Number of MICP Treatments	Immersion Time (h)	Curing Temperature (°C)	Total WD Cycles	Treatment Method
0	0	14	6	25 ± 1	30	Distilled water (DW) (fully submerged)
1	((0.5) 15)	14	6	25 ± 1	30	
2	((1.5) 15)	14	6	25 ± 1	30	
3	((3) 15)	14	6	25 ± 1	30	
4	((5) 15)	14	6	25 ± 1	30	
5	((10) 15)	14	6	25 ± 1	30	
6	((20) 15)	14	6	25 ± 1	30	

**Table 3:** Testing conditions used in this study for durability analysis (ASW cases).

Testing Cases	Fiber Content ((%) mm)	Number of MICP Treatments	Immersion Time (h)	Curing Temperature (°C)	Total WD Cycles	Treatment Method
0	0	14	6	25 ± 1	30	Artificial seawater (ASW) (fully submerged)
1	((0.5) 15)	14	6	25 ± 1	30	
2	((1.5) 15)	14	6	25 ± 1	30	
3	((3) 15)	14	6	25 ± 1	30	
4	((5) 15)	14	6	25 ± 1	30	
5	((10) 15)	14	6	25 ± 1	30	
6	((20) 15)	14	6	25 ± 1	30	

## Data Availability

The data presented in this study are available on request from the corresponding author.
